# Supplementary material for: Structural insights into functional properties of the oxidized form of cytochrome c oxidase
Source: Nat Commun. 2023 Sep 16;14:5752. doi: 10.1038/s41467-023-41533-x (PMC10505203; doi:10.1038/s41467-023-41533-x)
Supplement: Supplementary file 1 — Supplementary Information [file 41467_2023_41533_MOESM1_ESM.pdf]

Supplementary Information

Structural insights into functional properties of the oxidized form of cytochrome *c* oxidase

Izumi Ishigami[1], Raymond G. Sierra[2], Zhen Su[2,3], Ariana Peck[2], Cong Wang[2], Frederic Poitevin[2], Stella Lisova[2], Brandon Hayes[2], Frank R. Moss III[2,†], Sébastien Boutet[2], Robert E. Sublett[2], Chun Hong Yoon[2], Syun-Ru Yeh[1,*], and Denis L. Rousseau[1,*]

1. Department of Biochemistry, Albert Einstein College of Medicine, Bronx, NY 10461 USA
2. Linac Coherent Light Source, SLAC National Accelerator Laboratory, Menlo Park, CA 94025 USA
3. Department of Applied Physics, Stanford University, Stanford, CA 94305 USA

The PDF file includes:

Suplementary Figures 1 to 3
Supplementary Table 1
Suplementary References

[†]Current address: Frank R. Moss III, Altos Labs, Redwood City, CA 94065
*Address correspondence to: syun-ru.yeh@einsteinmed.edu or denis.rousseau@einsteinmed.edu

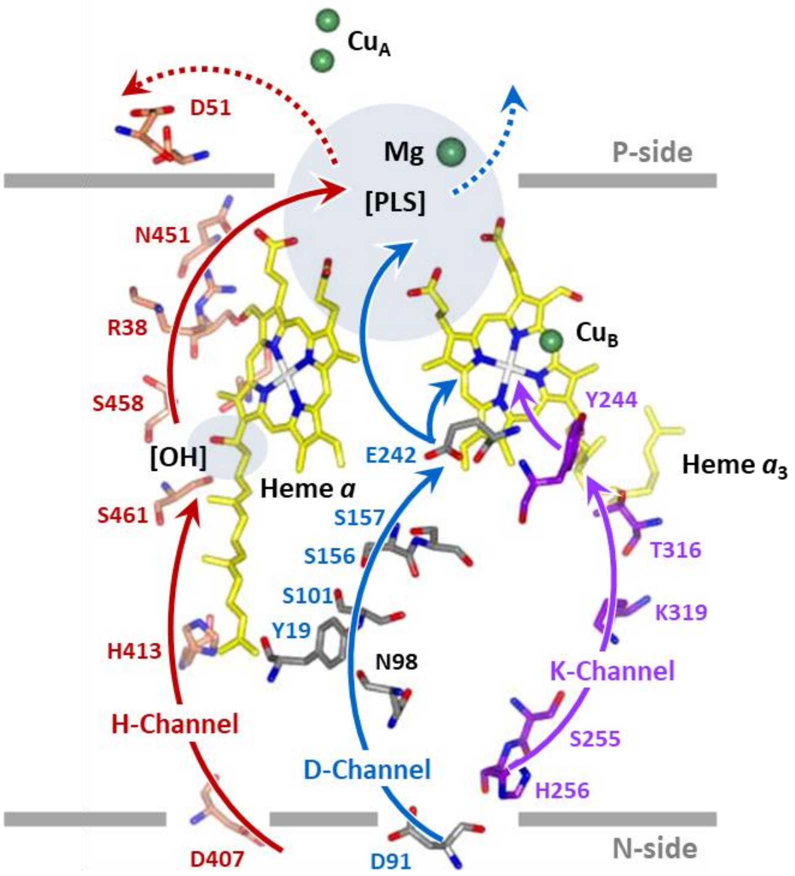

Supplementary Fig. 1. Proton transfer channels in bCcO. Based on structural and mutagenesis studies of bacterial enzymes, two proton transfer channels, the D and K-channels, have been proposed in C*c*O[1]. The D-channel, starting at D91 (bC*c*O numbering) and ending at E242 (which serves as a diverter valve), delivers either substrate protons to the BNC during the oxidative phase of the catalytic cycle, or pumped protons to the PLS for subsequent translocation to the P-side of the membrane. The K-channel, starting at H256 and ending at Y244, delivers substrate protons to the BNC during the reductive phase of the catalytic cycle. An additional channel, the H-channel, starting at D407 and ending at D51, was proposed in bC*c*O, which translocates pumped protons to the P-side of the membrane *via* the PLS, as gated by either a water pool[2] or the hydroxide group of the farnesyl sidechain (indicated as [OH])[3] of heme *a*. The role of the H-channel, however, remains controversial as it is not conserved in bacterial CcOs[4,5].

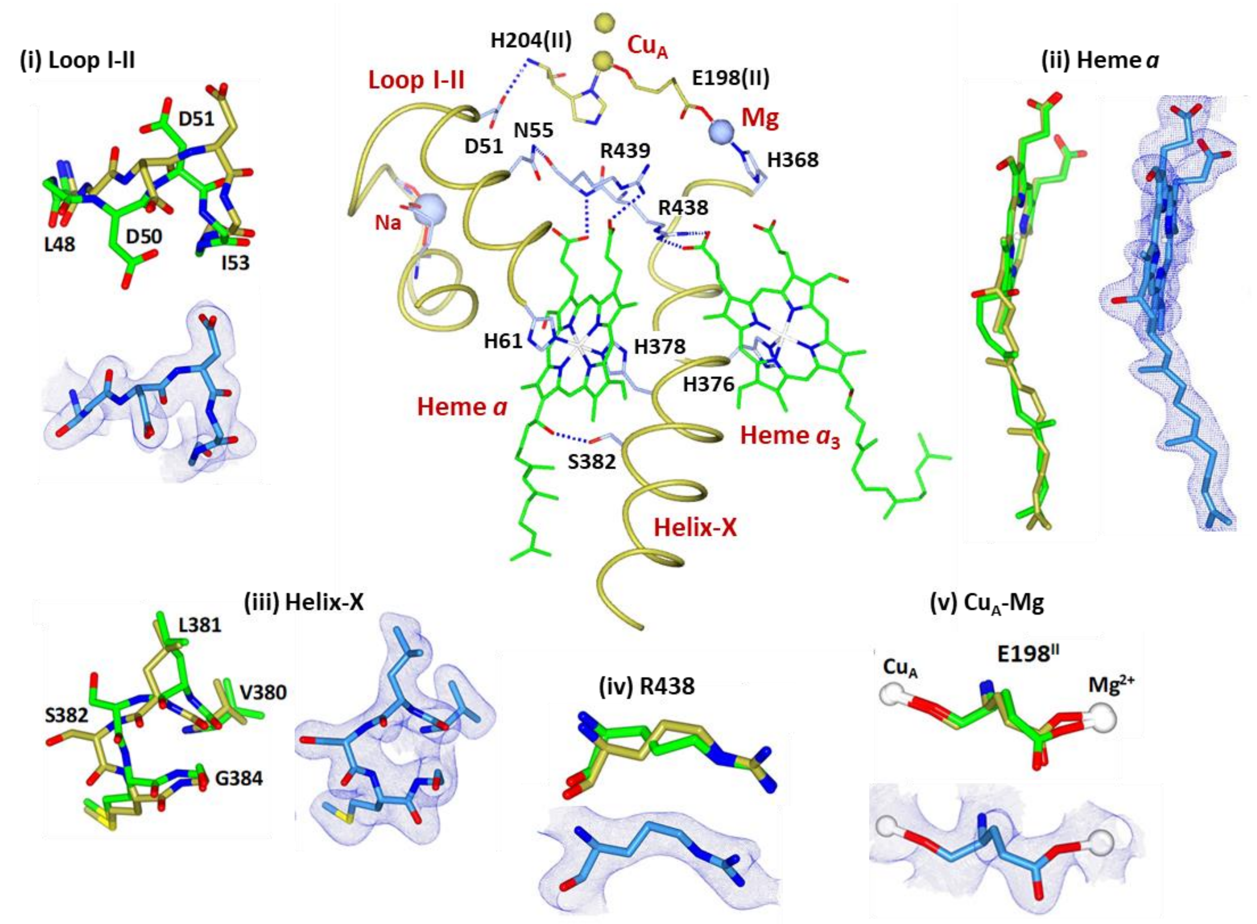

Supplementary Fig. 2. SFX structure of the O derivative of bC*c*O in the redox sensitive regions. Inset (i)-(v) show the structural comparison of the oxidized enzyme determined with synchrotron radiation (**O***) (in yellow) and reduced enzyme (**R**) (in green) in the redox sensitive regions. The $2F_O$-F_C electron density map of **O**, contoured at 1.5 σ, is shown in each inset to demonstrate that the structure of **O**, consistent with that of **O***, is in the oxidized state. The PDB IDs of the **O*** and **R** are 7TIE and 7THU, respectively.

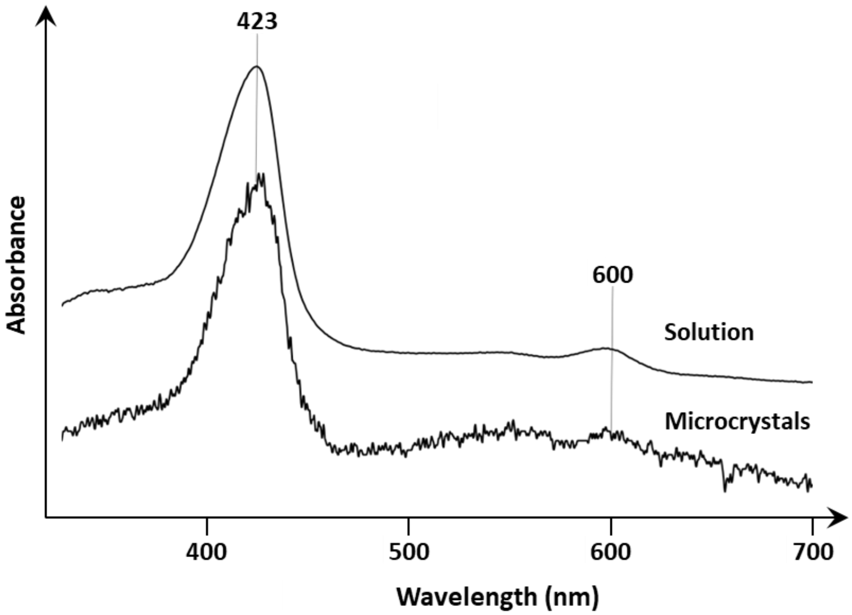

Supplementary Fig. 3. UV-Vis absorption spectra of oxidized bC*c*O in the free solution phase and in the microcrystalline state. The Soret maximum at 423 nm indicates that the bC*c*O samples are in the same fast (active) form.[6]

Supplementary Table 1. Crystallographic data collection and refinement statistics (molecular replacement).

	8GCQ
**Data collection**	
Wavelength(Å)	1.24
Temperature (K)	293
beamline	SLAC LCLS MFX
Space group	$P\ 2_1\ 2_1\ 2_1$
Cell dimensions	
a, *b*, *c* (Å)	178.60, 189.50, 211.10
α, β, γ (°)	90.00, 90.00, 90.00
Resolution (Å)	33.00-2.38(32.80-2.38) *
R_{sprit}	0.2296(0.6388)
$I / \sigma I$	4.59(1.78)
Completeness (%)	100(100)
Redundancy	2742(2286)
**Refinement**	
Resolution (Å)	35.00-2.38
No. reflections	270,854
R_{work} / R_{free}	0.232 / 0.266
No. atoms	31,465
Protein	28,514
Ligand/ion	2,350
Water	601
B-factors	34.79
Protein	32.42
Ligand/ion	64.39
Water	31.74
R.m.s. deviations	
Bond lengths (Å)	0.0114
Bond angles (°)	1.742
Ramachandran statistics (%)	
Favored	89.24
Allowed	9.73
Outliers	1.03

*Values in parentheses are for highest-resolution shell.

Supplementary References

1 Wikstrom, M., Krab, K. & Sharma, V. Oxygen Activation and Energy Conservation by Cytochrome c Oxidase. *Chemical reviews* **118**, 2469-2490, doi:10.1021/acs.chemrev.7b00664 (2018).

2 Shimokata, K. *et al.* The proton pumping pathway of bovine heart cytochrome c oxidase. *Proceedings of the National Academy of Sciences of the United States of America* **104**, 4200-4205, doi:10.1073/pnas.0611627104 (2007).

3 Ishigami, I. *et al.* Snapshot of an Oxygen Intermediate in the Catalytic Reaction of Cytochrome c Oxidase. *Proc Nat Acad Sci (USA)* **116**, 3572-3577 (2019).

4 Qin, L. *et al.* Redox-dependent conformational changes in cytochrome C oxidase suggest a gating mechanism for proton uptake. *Biochemistry* **48**, 5121-5130, doi:10.1021/bi9001387 (2009).

5 Yoshikawa, S. & Shimada, A. Reaction mechanism of cytochrome C oxidase. *Chemical reviews* **115**, 1936-1989, doi:10.1021/cr500266a (2015).

6 Baker, G. M., Noguchi, M. & Palmer, G. The reaction of cytochrome oxidase with cyanide. Preparation of the rapidly reacting form and its conversion to the slowly reacting form. *The Journal of biological chemistry* **262**, 595-604 (1987).
